# Size and position of the optic disc crescent in a white European population with myopia

**DOI:** 10.1111/opo.13018

**Published:** 2022-06-20

**Authors:** David Hill, Rebekka Heitmar, Nicola S. Logan

**Affiliations:** ^1^ Specsavers Newmarket UK; ^2^ School of Applied Sciences, Centre for Vision across the LifeSpan (CVLS) University of Huddersfield Huddersfield UK; ^3^ School of Optometry Aston University Birmingham UK

**Keywords:** crescent, myopia, optic nerve head, peripapillary atrophy

## Abstract

**Significance:**

One of the first clinically observed changes in the retina with progressing myopia is in the form of optic disc crescents. If such a change is predictive of myopia progression, it could aid in myopia management interventions to target those at greatest risk of progression and subsequent ocular morbidity.

**Purpose:**

To investigate the type, dimension and appearance of optic disc crescents and how they relate to the level of myopia.

**Methods:**

Retrospective data collection analysing retinal photographs of healthy children and adults with a refractive error of ≤−0.50 D sphere and astigmatism ≤2.00 D. Crescent location, maximum crescent width and vertical disc diameter were measured from retinal images of right eyes only.

**Results:**

Four‐hundred eyes with a mean spherical error (SER) of −0.50 to −14.00 D (aged 7–81 years) were included (83.5% exhibited a discernible crescent). Mean (SD) maximum crescent width was 0.24 (0.24) mm. Univariate analysis showed a significant correlation between crescent width and age (*r* = 0.26, *p* < 0.001). SER was correlated with crescent width when controlling for age (*r* = −0.45, *p* < 0.001) and to the ratio of crescent width to vertical disc diameter (*r* = −0.43, *p* < 0.001). Temporal crescents were the most frequently observed (74%), followed by inferior temporal crescents (17%). One‐way between‐groups analysis of variance showed a significant difference between crescent locations (*F* = 5.2, *p* < 0.001). *Post‐hoc* analysis revealed significant differences in SER between those with no crescent versus an inferior‐temporal crescent, as well as differences between those with temporal versus inferior‐temporal crescents. Other crescent locations did not differ significantly in the level of myopia. Participants not exhibiting a crescent had the lowest level of myopia (mean [SD] −3.03 [1.97)] D), while those with inferior temporal crescents had a mean (SD) SER of −5.01 (2.37) D.

**Conclusion:**

In this white European population, higher levels of SER were associated with increasing crescent size. Eyes with inferior temporally located crescents were more myopic.

**Key points**

- This practice‐based study assessed the relationship between the level of myopia and optic disc crescent prevalence, size and location.
- Six patterns of crescent were observed. Temporal crescents were the most prevalent; however, inferior temporal crescents were associated with a higher degree of myopia and may be a potential marker for risk of greater myopic progression.
- Twenty per cent of the variance in crescent size was explained by the level of myopia with no influence of age.

## INTRODUCTION

The optic nerve head (ONH) is associated with many pathologies,^1^ and is arguably the key fundus feature observed in a retinal examination. Digital fundus images are now a standard part of routine eye examinations conducted by UK optometrists.^2^ They provide the practitioner with a permanent record and facilitate the qualitative and quantitative assessment of retinal structures such as vessel calibres, as well as structural dimensions such as disc and cup size and crescents. Changes occurring over time and follow‐up measurements to track treatment responses can be conducted more precisely with the use of images rather than by visual inspection.

A typical myopic ONH may be tilted, have an elongated vertically ovoid shape, shallow cupping and display low contrast between the neuro‐retinal rim and cup; however, the most frequently observed feature is a myopic crescent.^3^, ^4^ Crescents seen adjacent to the optic nerve head have been described in the literature using several different terms including peri‐ or para‐papillary atrophy (PPA), myopic crescent, temporal crescent, sickle or conus myopicus.^5^ It has been suggested that eyes with physiological myopia should not have a crescent unless congenital and the crescent is no greater than 10% of the disc diameter.^4^ Congenital crescents are described as being a result of local developmental changes and are usually stationary, whereas eyes with moderate myopia (<6.00 D) and no other visible retinal changes typically have a crescent which may increase in size as myopia progresses.^3^, ^6^ When the crescent size increases, the sharply demarcated borders can become less distinct, which can increase the size of the blind spot^7^ when assessing the ONH area using microperimetric methods. The myopic crescent seen at non‐pathologic levels of myopia is in most instances temporal, but can also occur in other positions.^8^ The colouration of the crescent depends on the patient's pigmentation and the structures, which are misaligned at the disc margin.

A significant increase in the prevalence of myopia has been reported worldwide,^9^, ^10^ with the highest prevalence in the higher income countries of South East Asia.^11^ The increasing prevalence, in conjunction with an ageing demographic, will soon magnify the impact of myopia‐associated ocular morbidity. Optic nerve crescents may result from excessive stretching of the ocular tissues as a consequence of axial growth, and the presence of a crescent (and increasing crescent size over time) may be indicative of an eye at increased risk of ocular morbidity. Hence, the presence of a crescent may be a clinical biomarker to predict future myopic progression.^12^ Crescents have previously been investigated in Asian populations^3^, ^13^ but rarely in white European groups. While it is common in myopia research to measure axial length and to use cycloplegic refraction, we adopted a translational approach in this study using standard UK primary care optometry clinical practice tools to identify, measure and describe the location, dimensions, and associations of optic disc crescents with the level of myopia in a predominantly adult white European (located in the UK) community‐based population.

## METHODS

### Participants

Following favourable ethical review by the Aston University Research Ethics Committee (REC No 642) and compliant with the tenets of the Declaration of Helsinki, the records of 400 myopic patients who had previously attended for a routine eye examination at a community optometric practice in a semi‐rural area of the United Kingdom (East Anglia) were assessed retrospectively. Inclusion criteria were spherical refraction of ≤−0.50 D and astigmatism not greater than 2.00 D. Eyes with known ocular pathology as stated on the patient record or previous eye surgery were excluded. All subjects had undergone 45‐degree retinal photography (macula centred) using a Nidek AFC‐210 non‐mydriatic camera (Nidek.com). Refractive status was obtained by non‐cycloplegic subjective refraction. Standard clinical optometric practice protocols for assessment of refraction were followed, that is objective refraction refined by distance fixation subjective refraction. Participants were identified from a database enquiry to search all records which met the refractive inclusion criteria. Once identified, their retinal photographs were located and included only if they had sufficient image quality as described elsewhere.^14^ In brief, images required a minimum score of 4 or above (based on the scale used by Lamirel et al.^14^) and the ONH, including any crescent, had to be in focus for inclusion. With each subsequent record selected, this process was continued until the required number of records, as based on our sample size calculation, was reached. For overview purposes, we plotted the presence of crescents/no crescents across five age groups (i.e., <18 years, 19–35 years, 36–50 years, 51–65 years, >66 years) and refractive error groups: −0.5 to −1.99 D, −2.00 to −3.99 D, −4.00 to −5.99 D, −6.00 to −7.99 D, ≤−8.00 D.

### Sample size estimation

Sample size was estimated using G*power software (Heinrich Heine University, hhu.de).^15^ A sample size of 138, based on Pearson's correlation with a moderate effect size (f 0.3) and power of 0.95 was calculated. To allow for ‘record’ exclusion based on the inclusion criteria described above (i.e., the presence of ocular pathology and/or ocular surgery as noted on the record and poor retinal image quality), and to allow meaningful sub‐groups analyses, we chose a sample size of 400 participants to be identified from records.

### Image analysis

All retinal images were assigned a unique participant ID and were analysed by a single observer (DH) masked to the level of refractive error. The vertical ONH (disc) diameter (VDD) and maximum crescent width (CW) were measured in millimetres (mm) using the retinal cameras' inbuilt Navis‐Lite software (Image Wizard version 1.6, Nidek.com). The absolute measurements were corrected for ocular magnification using the method described by Bengtsson.^16^ In brief, the following correction factor was used to correct dimensional measurements: (1–0.017 G), where G is the spectacle refraction.

**FIGURE 1:**
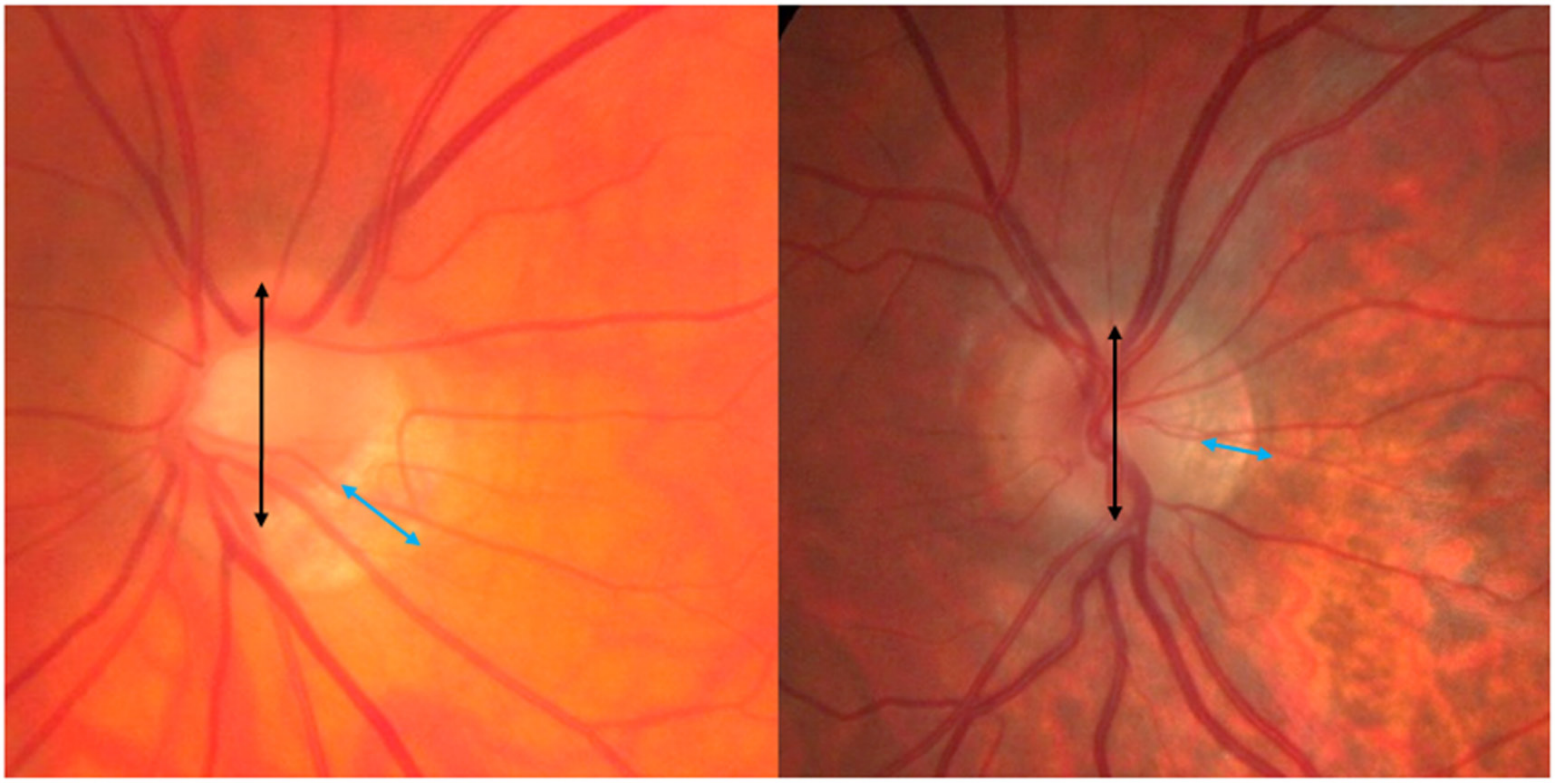
Fundus photograph showing the measurements taken using the Navis‐lite tools: The black line indicates the vertical disc diameter (VDD) and the blue line the maximum crescent width (CW).

The position of the maximum crescent width was recorded as follows: six patterns were identified as described in previous reports^8^, ^16^ detailing their relative location to the optic disc (nasal, inferior, temporal and nasal, inferior temporal, temporal and halo). In addition, a randomly selected subset of 25 images (out of the entire sample of 400) was measured twice to assess intra‐observer variability, and 10 randomly selected images (out of the entire sample of 400) were measured 10 times each to allow calculation of the coefficient of variation (CoV). When determining the ONH dimensions (i.e., neuro‐retinal rim, cup, disc) the area within the scleral ring of Elschnig was considered as the optic disc, and the crescent was measured from the edge of the scleral ring to the most distant part of the pigmentary change (Figure 1). This process was completed manually by one examiner (DH) masked to the level of refraction, and repeatability of technique was assessed as detailed below.

The quotient of the maximum CW to the VDD was calculated as described previously^17^, ^18^ to provide a crescent index independent of ocular magnification. This index is useful in determining changes in crescent width as the vertical disc height has previously been shown to be stable over time.^19^

### Statistical analysis

All data were analysed using SPSS (IBM SPSS Statistics for Windows version 23, ibm.com). Repeatability of disc measurements was assessed using Bland–Altman analyses and calculation of the mean CoV. A strong correlation was observed between the right and left eyes for SER (*r* = 0.92, *p* < 0.001) and for vertical disc diameter (*r* = 0.81, *p* < 0.001). An independent samples *t*‐test was carried out to compare SER, VDD and CW for each eye. All three measures showed no differences (*p*‐values = 0.73, 0.91 and 0.62, respectively). Therefore, we performed all analyses using data from the right eye only.

## RESULTS

**FIGURE 2:**
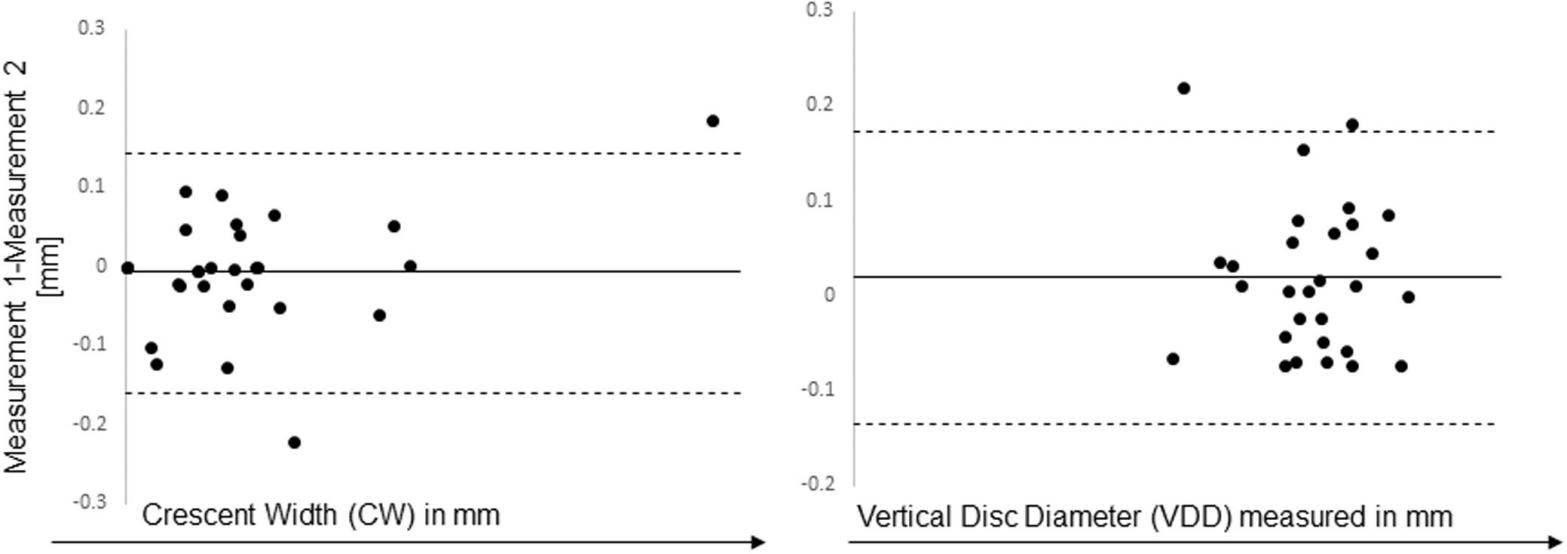
Bland–Altman plots for crescent width (CW) and vertical disc diameter (VDD) showing good agreement of measures.

Bland–Altman and COV analyses showed good agreement and reproducibility for CW and VDD (Figure 2). The average COV, calculated across 10 images where each image was measured 10 times, was 1.95% for the VDD and 9.8% for CW. Bland–Altman plots showed a bias close to zero and narrow limits of agreement.

Records extracted from all participants (*n* = 400) consisting of 290 females (73%) and 110 males (27%), with an age range of 7 to 81 years at the time of image capture (mean (SD) age 41 (16.24) years). The average (SD) level of myopia (SER) was −3.74 (2.32) D (range: −0.50 to −14.00 D). Results of VDD and the maximum CW are detailed in Table 1. While 333 (83%) eyes exhibited a crescent at the disc, 67 (16.75%) did not.

**TABLE 1:** Optic nerve head metrics

Parameter, *n* = 333	Mean	Median	Standard deviation	Range
VDD (mm)^a^	1.75	1.75	0.26	0.90–2.70
CW (mm)^a^	0.29	0.21	0.23	0.025–1.85
Max CW/VDD	0.17	0.12	0.15	0.01–1.21

Abbreviations: CW, crescent width; VDD, vertical disc diameter.

^a^ Corrected for ocular magnification.

**FIGURE 3:**
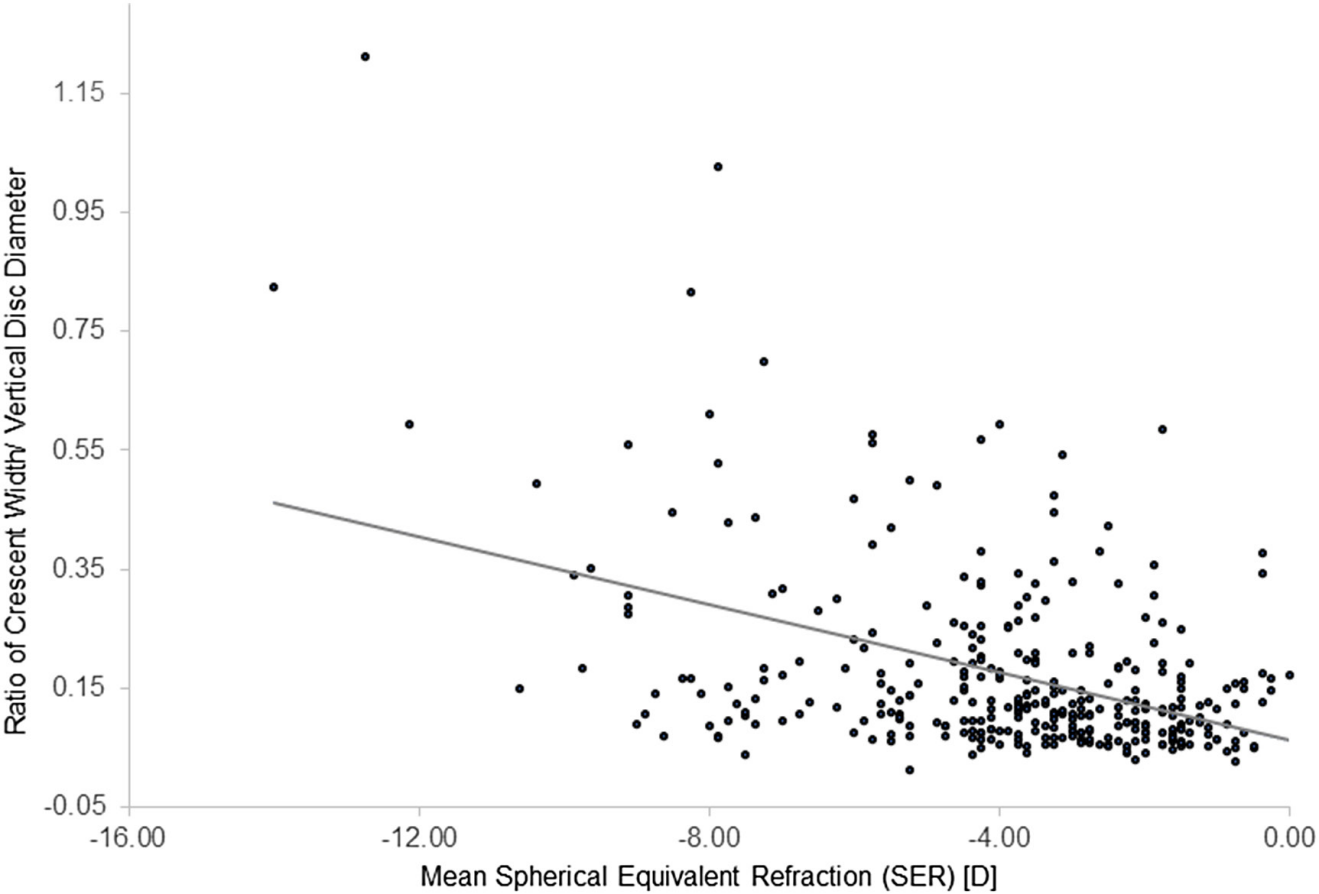
Scatterplot showing ratio of widest crescent width (CW) to vertical disc diameter (VDD) plotted against myopia (SER) for the right eye.

Univariate analysis showed the quotient of the CW to VDD to be significantly correlated with SER (*r* = −0.44, *p* < 0.001) (Figure 3).

**FIGURE 4:**
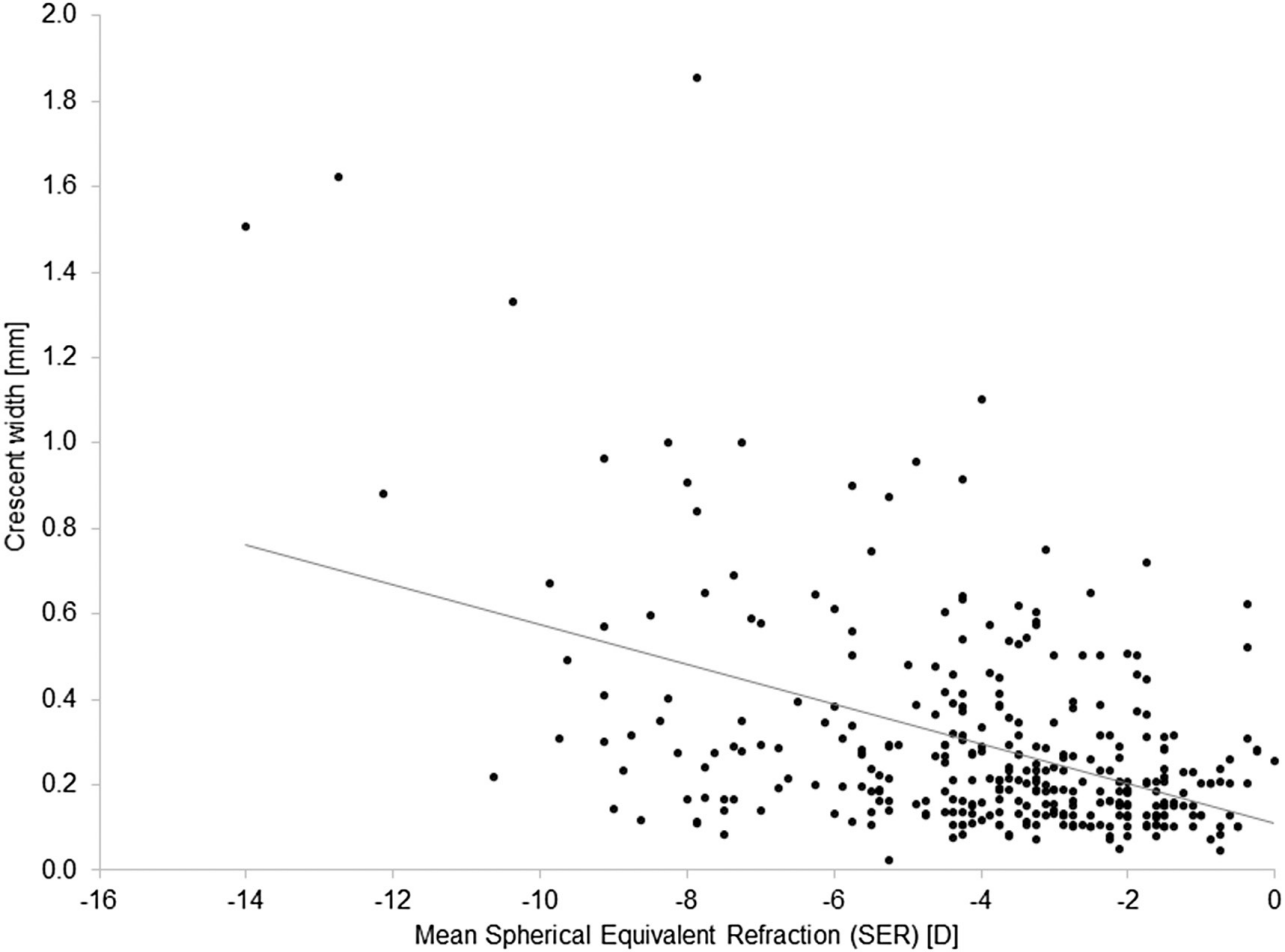
Scatter plot showing correlation between crescent width (CW) and mean spherical equivalent.

Multivariate modelling controlling for age, sex and SER explained 24.4% of the variance in the quotient of crescent width to vertical disc diameter. Of the three variables, SER (*β* −0.43, *p* < 0.001) and age (*β* 0.25, *p* < 0.001) were significant predictors while sex was not (*β* −0.04, *p* = 0.40). Simple linear regression analysis for the absolute measure showed the crescent width to increase with the level of myopia (SER) (*r* = −0.44, *p* < 0.001). Partial correlation analysis, controlling for age, showed increasing CW with increasing levels of myopia (*r* = −0.45, *p* < 0.001) (Figure 4).

**FIGURE 5:**
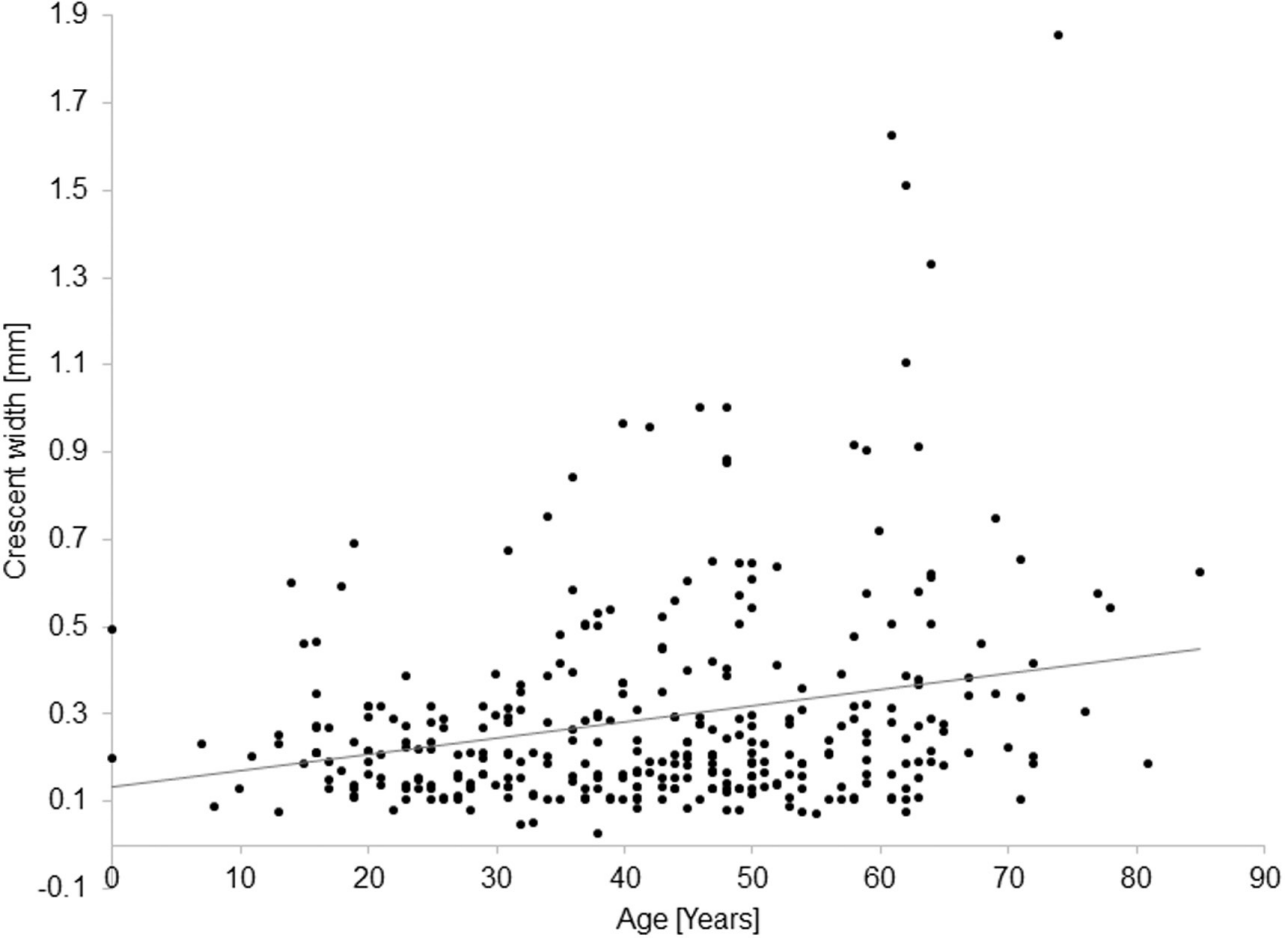
Age plotted against the corrected crescent width for the right eye.

There was no relationship between age and SER (*r* = −0.01, *p* = 0.81). The two measures of crescent size (maximum CW (Figure 5) and the ratio of the maximum CW to VDD) increased with age (*r* = 0.26, *p* < 0.001 and *r* = 0.25, *p* < 0.001). No difference in the crescent size was observed between men and women.

The position of the peripapillary crescent was most frequently temporal (74%) (Table 2) followed by inferior temporal (17%). The eyes with no crescent had a mean SER of −3.03 D, those with an inferior temporal crescent −5.01 D and in eyes with a temporally located crescent −3.50 D. A one‐way between‐groups analysis of variance was conducted to explore the relationship between myopia (SER) and position of the crescent. A statistically significant difference across the groups was found, *F* = 5.2 *p* < 0.001. *Post‐hoc* comparisons using Tukey's honestly significant difference (HSD) test indicated that the level of SER was significantly different between those with no crescent and an inferior‐temporal crescent and between temporal and inferior‐temporal crescents. The other comparisons did not differ significantly. The largest crescents were of the temporal‐nasal pattern (see Table 2) and the smallest crescents were located on the temporal margin of the ONH.

**TABLE 2:** Distribution of crescent positions

Crescent position	*n*	Mean SER (SD)	Mean crescent width (SD) (mm)
None	67	−3.03 ± 1.97	—
Halo	18	−4.64 ± 3.40	0.47 ± 0.46
Temporal	249	−3.50 ± 2.14	0.23 ± 0.17
Inferior temporal	56	−5.01 ± 2.37	0.38 ± 0.04
Nasal and Temporal	2	−4.50 ± 3.71	0.49 ± 0.97
Inferior	5	−5.32 ± 2.68	0.40 ± 0.18
Nasal	3	−6.20 ± 1.75	0.45 ± 0.31

*Note:* The mean spherical error (SER) for each position of the crescent and mean crescent width are shown.

**FIGURE 6:**
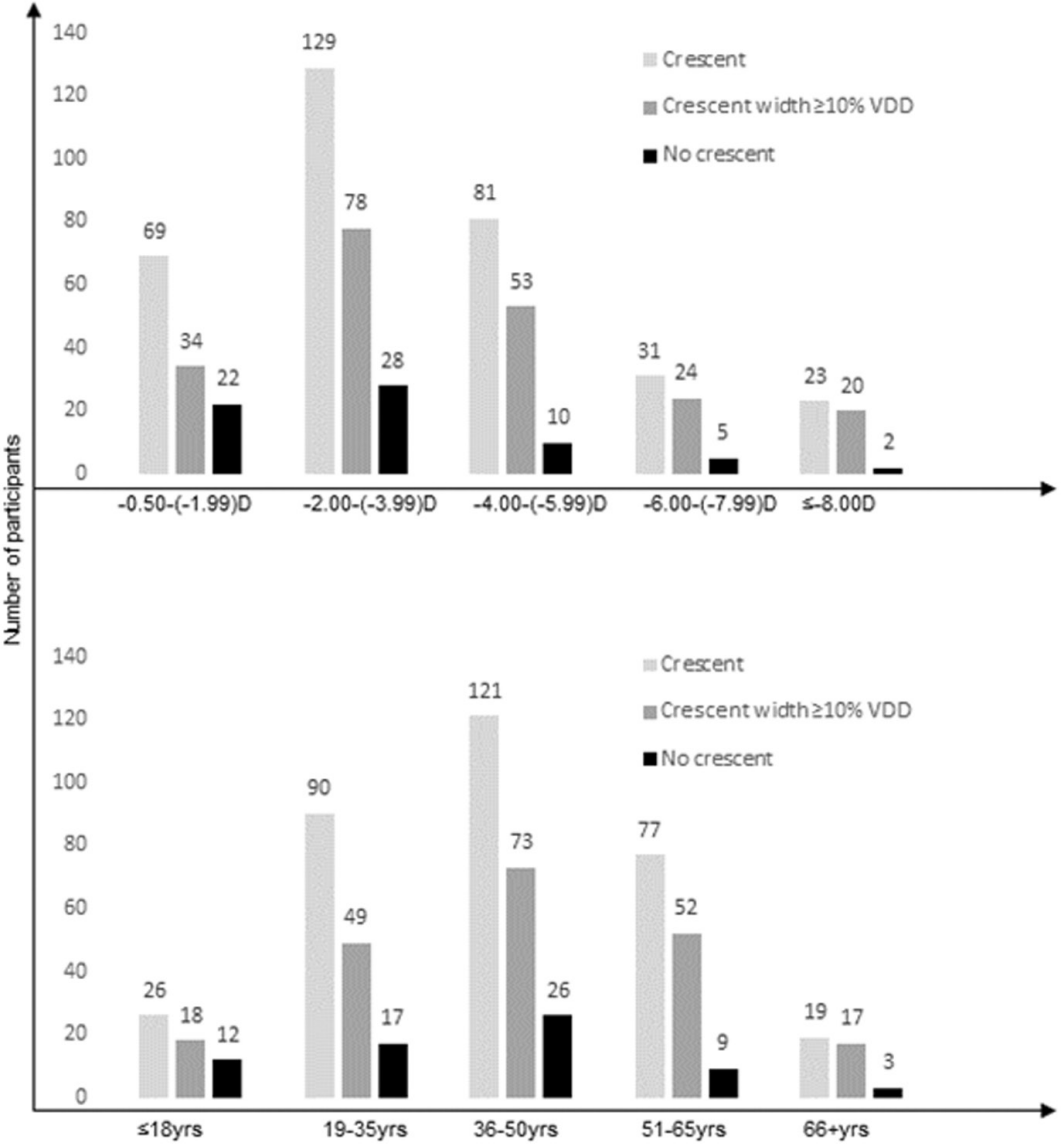
Distributions of no crescent (black bar), crescents widths of equal or greater than 10% of the vertical optic disc diameter (dark grey bar) and any crescent (light grey bar) across refractive error and age groups.

Figure 6 provides an overview of the distribution of crescents as a function of refractive error and age. Ninety‐two percent of eyes in the highest category of myopia had a crescent compared to only 75% in the group with the lowest level of myopia. When those with a crescent <10% of the disc diameter were excluded, 80% in the highest category of myopia were recorded as having a crescent compared with only 39% in the lowest category. When comparing age groups, we allocated five categories: ≤18 years of age, 19–35 years of age, 36–50 years of age, 51–65 years of age and >66 years of age. Groupings were chosen to have data sets allowing for comparison with other reports, and to reflect age boundaries at which age‐related changes are most commonly present. CW in the two oldest age categories were comparable, as were those of the three youngest. The widths of the two oldest categories were wider than the younger groups (oldest group compared to the three youngest groups all *p* < 0.01). A two‐way analysis of variance exploring the impact of myopia and age on crescent size was also significant (*F*(15) = 3.77; *p* < 0.0001).

## DISCUSSION

This study set out to document the prevalence, location and size of optic nerve crescents and associations with the level of myopia and age in a white European population. Cross‐sectional contemporary UK data from a white European optometric practice show that optic disc crescent and size are linked with the level of myopia and age.

Eighty‐three per cent of eyes had a discernible crescent, which is in agreement with previous work by Jonas et al.^8^ but somewhat higher than the 59% reported in a white European population in Rotterdam.^19^ Reasons for the discrepancy may be found in the fact that the Rotterdam study included hyperopes and excluded anyone with myopia >8.00 D, along with differences in crescent definition. A study of young Japanese myopes, using similar methods to the current study, reported crescents to be present in two‐thirds of eyes,^13^ which agrees with our observations when older eyes are excluded. In those eyes with crescents, we observed six distinct patterns. Temporally located crescents were the most frequently occurring (74%), followed by inferior temporal (16.8%). Curtin^4^ described 12 patterns of crescent by location using direct ophthalmoscopy, and similarly found temporally located crescents to be the most frequent (62%), with inferior‐temporal crescents occurring in 2.3% of cases. The findings of the present study, therefore, show good reproducibility with previous work.

One‐way, between‐groups analysis of variance exploring the differences between the level of myopia (SER) depending upon the position of the crescent showed that the level of SER was only significantly different between those with no crescent (−3.03 ± 1.97 D) and an inferior‐temporal crescent (−5.01 ± 2.37 D), as well as between temporal (−3.50 ± 2.14 D) and inferior‐temporal crescents (−5.01 ± 2.37 D). While those with inferior only and nasal only crescents had similar levels of myopia to those exhibiting inferior‐temporal crescents (−5.32 ± 2.68 D and −6.20 ± 1.75 D, respectively), the lack of difference to those without crescents (−3.03 ± 1.97 D) can be explained by the difference in group size (see Table 2).

Perkins^20^ reported that eyes with more than 4.00 D of myopia usually had a crescent, and Grosvenor^21^ observed that children with myopia of 3.00 D invariably had crescents, whereas those with ≤1.50 D of myopia tended not to. In this study, only 8% of eyes in the highest category of myopia (<−8.00 D) did not have a crescent. The high prevalence of optic disc crescents in those with low myopia (>2.00 D) may be due to the inclusion of congenital crescents and the significant number of older participants in our group, whose myopia may be due to lenticular changes and the fact that age has also been associated with peripapillary changes.^22^ Excluding small crescents (<10% of the disc diameter, as they are likely to be congenital in nature) and re‐evaluating the prevalence of crescents with age, the prevalence of crescents increased by 100% between the youngest and oldest groups. Some of this variance may be explained if the myopia amongst those in the lower categories of myopia were still progressing, especially if they were also younger. However, the level of myopia showed no relationship with age in this study (*r* = −0.01, *p* = 0.81).

In this translational study, the level of myopia explained 20% of the variance in crescent size, with age not altering the relationship (*r* = −0.44, *p* < 0.001). A similar, albeit weaker relationship was reported by Marsh‐Tootle and colleagues,^23^ which may be due to their sample being more ethnically diverse than ours. They reported a relationship between crescent width and optic disc tilt, which was dependent on ethnicity. Furthermore, they suggested that their data supports Curtin's proposal that the presence of a crescent, rather than the axial length, is an indicator that the eye length is excessive. The size of the crescent varied according to its location. The widest crescents were halo, temporal‐nasal and nasal. However, the number of eyes with these patterns was small (13, 2 and 3, respectively) and may not be solely myopia related but a consequence of other factors. Temporal crescents were smallest in size and were associated with a lower SER inferring a more regular eye shape. Curtin observed halo‐type crescents in eyes with the longest axial lengths.^24^ In addition to absolute measures, we used the ratio of the maximum CW to the VDD to negate the need for magnification correction. This ratio was found to be significantly correlated with SER (*r* = −0.43, *p* < 0.001), which agrees with Hyung's^18^ findings in a slightly younger group of Koreans (*r* = −0.53, *p* < 0.001), as well as those reported by Kim et al. in children.^17^

Optic nerve crescents in myopic eyes may be the result of the response to excessive tension in the sclera, choroid and retina.^25^, ^26^ The presence of a crescent may indicate an eye is at increased risk of morbidity in terms of myopic progression,^27^ development of myopic maculopathy^28^ and open‐angle glaucoma.^29^ Many of the retinal changes seen in the myopic eye appear to develop once the progression of myopia has ceased.^12^ Our finding of larger crescents in older myopes (*r* = 0.26, *p* < 0.001) supports this and is in agreement with previous reports.^22^, ^30^ Longitudinal review^17^ has shown the progression of childhood myopia, regarding both SER and axial elongation, results in enlargement of the crescent over a relatively short follow‐up period of 38 months. Nakazawa^6^ presented serial disc images of 10 Japanese low and moderate young adult myopes showing crescent formation as myopia progressed. Healey et al.^31^ investigated the inheritance of peripapillary changes in 506 pairs of adult twins. The prevalence of peripapillary changes was similar between monozygotic and dizygotic twins, indicating little or no genetic association, although the heritability was high (75%).

If the size of the optic disc is greater and the crescent is wider with higher levels of myopia, then the crescent should also be wider in eyes with larger discs. This is the conclusion suggested by Jonas,^1^ but conflicts partially with the observations of the current study. It is agreed that higher myopia is associated with greater PPA. However, some cases with high myopia and/or tilted discs were found to have smaller discs but larger areas of PPA. Crescent width was found to decrease as the VDD increased (*r* = −0.24, *p* < 0.001), further supporting this observation. Even in the higher levels of myopia, a crescent was often absent. In this optometry practice population, axial length measurements were not available, and therefore, it is not possible to say whether the myopia was due to refractive factors rather than an increase in axial length.

Myopic fundus changes appear dependent on the tensions in the three tissues^32^ and non‐uniform re‐modelling of the posterior sclera.^24^ The findings from this study show that age and SER are predictive of the crescent dimensions. This is expected to some extent, as with increasing myopia the frequency of a crescent is more likely. Age in this context explains not only the longstanding ‘stretch’ but is also part of the magnitude of elongation and longstanding stress, as well as age‐related factors involved in tissue mechanics.

Limitations to this translational approach are partly linked with its setting: SER was used to quantify the level of myopia as axial length measurements were not available. While axial length measures are desirable, not least to ascertain axial myopia, SER is highly correlated with axial length^33^ and allowing our findings to be directly accessible and translational to optometric clinical practice. We measured the crescent from the edge of the scleral ring of Elschnig to the furthest point of pigmentary change covering both the alpha and beta zones. Planimetry of fundus photographs remains a reliable method of assessing the disc and adjacent area.^22^ Although expert assessment of fundus photographs remains the gold standard,^34^ the subjective nature leads to the disadvantage of inter‐ and intra‐observation variability. Only one observer was used in this study, and the assessment of reliability showed a high level of repeatability. Despite adjusting the absolute ONH measures for ocular magnification, several assumptions and related measurement factors make the measures at best an approximation. As the size of the measures is similar to previous studies, and we used a ratio measure negating the need for magnification correction, we are confident that any error is small. Selection bias was minimised by selecting the participants in patient number order if they had clear fundus images.

In conclusion, we have documented the associations between myopic eyes and peripapillary appearance in a white European population. Higher myopia (SER) was associated with the presence of an optic disc crescent as well as larger and inferior‐temporally located crescents.

Monitoring the appearance of optic disc crescents from childhood using clinical imaging would allow study of whether crescent formation and change precedes and/or is capable of predicting the development of myopic ocular pathology.

## AUTHOR CONTRIBUTIONS

**David Hill:** Data curation (equal); formal analysis (equal); investigation (equal); methodology (equal); writing – original draft (equal); writing – review and editing (equal). **Rebekka Heitmar:** Methodology (equal); supervision (equal); validation (equal); writing – original draft (equal); writing – review and editing (equal). **Nicola S Logan:** Conceptualization (equal); methodology (equal); project administration (equal); resources (equal); supervision (equal); validation (equal); writing – original draft (equal); writing – review and editing (equal).

## CONFLICT OF INTEREST

The authors declare no conflicts of interest.
